# Flavonoids are determinants of freezing tolerance and cold acclimation in *Arabidopsis thaliana*

**DOI:** 10.1038/srep34027

**Published:** 2016-09-23

**Authors:** Elisa Schulz, Takayuki Tohge, Ellen Zuther, Alisdair R. Fernie, Dirk K. Hincha

**Affiliations:** 1 Max-Planck-Institut für Molekulare Pflanzenphysiologie, Am Mühlenberg 1, D-14476 Potsdam, Germany

## Abstract

In plants from temperate climates such as *Arabidopsis thaliana* low, non-freezing temperatures lead to increased freezing tolerance in a process termed cold acclimation. This process is accompanied by massive changes in gene expression and in the content of primary metabolites and lipids. In addition, most flavonols and anthocyanins accumulate upon cold exposure, along with most transcripts encoding transcription factors and enzymes of the flavonoid biosynthetic pathway. However, no evidence for a functional role of flavonoids in plant freezing tolerance has been shown. Here, we present a comprehensive analysis using qRT-PCR for transcript, LC-MS for flavonoid and GC-MS for primary metabolite measurements, and an electrolyte leakage assay to determine freezing tolerance of 20 mutant lines in two *Arabidopsis* accessions that are affected in different steps of the flavonoid biosynthetic pathway. This analysis provides evidence for a functional role of flavonoids in plant cold acclimation. The accumulation of flavonoids in the activation tagging mutant line *pap1-D* improved, while reduced flavonoid content in different knock-out mutants impaired leaf freezing tolerance. Analysis of the different knock-out mutants suggests redundancy of flavonoid structures, as the lack of flavonols or anthocyanins could be compensated by other compound classes.

Flavonoids are phenylpropanoids and a major class of secondary plant metabolites which are, unlike primary metabolites, not associated with plant survival and growth under standard controlled growth conditions^1^. Experimentally this has the advantage that mutants in this pathway show unaltered growth and development compared to wild type plants^2^. In *Arabidopsis*, at least 35 flavonol and 11 anthocyanin derivatives were detected in various tissues including leaves^3^,^4^,^5^,^6^,^7^, flowers^6^,^8^, roots^4^,^6^,^9^,^10^ and seeds^11^,^12^. The structural variety of the compounds is due to different modifications of the basic skeleton. In *Arabidopsis*, the flavonol aglycones (kaempferol, quercetin and isorhamnetin) can be mono-, di-, or tri-glycosylated by Glc, Rha or Ara, while the anthocyanin aglycone cyanidin can be additionally modified by xylosyl, sinapoyl, *p*-coumaroyl or malonyl residues.

The genes underlying the flavonoid pathway in *Arabidopsis* are mostly single-copy genes, making this species a convenient organism to investigate flavonoid biosynthesis^2^. Flavonoid biosynthesis genes include genes encoding transcription factors regulating the pathway and genes encoding different enzymes involved in the biosynthesis of the aglycones and their modifications^4^,^7^,^9^,^13^,^14^.

A variety of functions were reported for flavonoids ranging from flower and fruit pigmentation to regulation of polar transport of the phytohormone auxin and plant defense against pathogens and UV light. In addition, flavonoids accumulate in response to abiotic stresses such as drought, cold and high light intensity and are thought to be important for tolerance against these stresses^15^,^16^. In particular, low temperature strongly increases flavonoid content^17^,^18^,^19^,^20^, the abundance of enzymes involved in flavonoid biosynthesis^21^ and the expression of flavonoid biosynthesis genes^20^,^22^,^23^,^24^ in various plant species. Often a purple leaf phenotype, due to the accumulation of anthocyanins, is taken as a general indicator of plant stress exposure.

Plant species from temperate regions, among them *Arabidopsis thaliana*, increase in freezing tolerance during exposure to low, but non-freezing temperatures in a process termed cold acclimation^25^,^26^. This involves a massive reprogramming of the transcriptome, proteome and metabolome (see refs 27 and 28 for reviews) including flavonols and anthocyanins^20^. The content of different flavonols is correlated with leaf freezing tolerance in various *Arabidopsis* accessions and their crosses^19^,^20^ and in addition the expression of some flavonoid biosynthesis genes and freezing tolerance are correlated in diverse *Arabidopsis* accessions^20^,^23^. However, a direct causal relationship between the accumulation of flavonoids and freezing tolerance or the ability to cold acclimate has not been shown.

## Results

**Figure 1:**
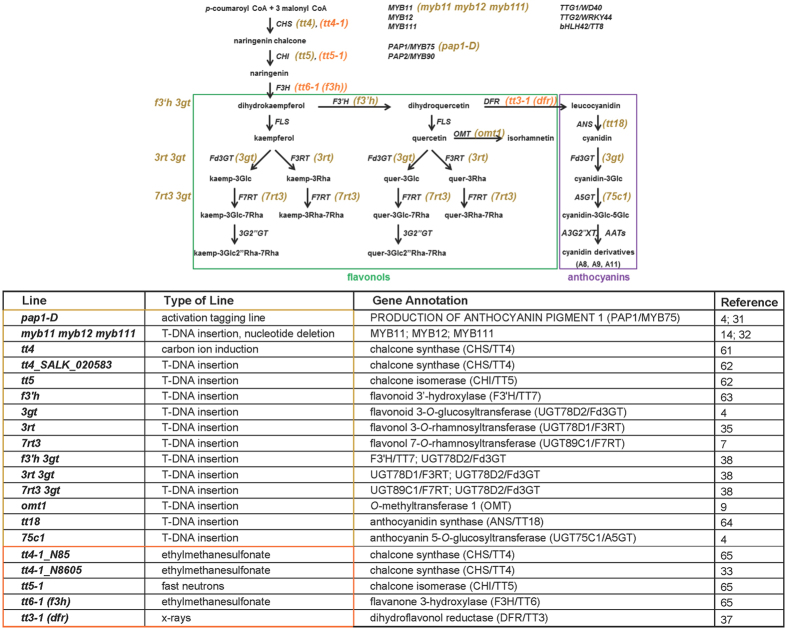
Flavonol and anthocyanin biosynthetic pathway indicating mutant lines used in this study. Genes and metabolites are shown in black, mutant lines in Col-0 background in yellow and in L*er* background in orange. Details of the mutant lines are listed in the bottom panel^4^,^7^,^9^,^14^,^31^,^32^,^33^,^35^,^37^,^38^,^61^,^62^,^63^,^64^,^65^. MYB11, MYB12, MYB111, R2R3 MYB transcription factors; TTG1/WD40, TTG2/WRKY44, transparent testa glabra; bHLH42/TT8, basic helix-loop-helix protein 42; PAP1/MYB75, PAP2/MYB90, production of anthocyanin pigment proteins 1 and 2; CHS/TT4, chalcone synthase; CHI/TT5, chalcone isomerase; F3H/TT6, flavanone 3-hydroxylase; F3′H/TT7, flavonoid 3′-hydroxylase; FLS, flavonol synthase; OMT1, *O*-methyltransferase 1; DFR/TT3, dihydroflavonol 4-reductase; ANS/TT18, anthocyanidin synthase; UGT78D2/Fd3GT, UGT78D1/F3RT, UGT89C1/F7RT, UGT75C1/A5GT, A3G2″XT, UDP-glucoronosyl/UDP-glucosyl transferase family proteins; AAT, anthocyanin acyl-transferase; TT, transparent testa.

We used a collection of 20 mutants in two different *Arabidopsis thaliana* accessions (Col-0 and L*er*) that cover all major regulatory and biosynthetic steps in flavonoid biosynthesis (Fig. 1) to investigate the role of flavonols and anthocyanins in plant freezing tolerance and cold acclimation. Nineteen of these mutants are single (15 lines), double (three lines) or triple (one line) gene knock-outs. In addition, we included the activation tagging line *pap1-D*, which exhibits constitutive overexpression of the gene *PAP1/MYB75* encoding the PRODUCTION OF ANTHOCYANIN PIGMENT 1 (PAP1) MYB transcription factor. The ko mutants showed no obvious phenotypic differences compared to the corresponding wild type plants under either non-acclimating or cold acclimating (14 days at 4 °C) conditions. This is in agreement with the finding that our cold acclimation conditions did not induce significant accumulation of reactive oxygen species (ROS)^29^ or ROS-related chilling stress that might require flavonoids for protection^15^,^30^. The *pap1-D* mutant, on the other hand, exhibited a purple leaf phenotype due to the activation of the whole flavonoid biosynthetic pathway^31^.

### Effects of mutations on the expression of flavonoid biosynthesis genes

**Figure 2:**
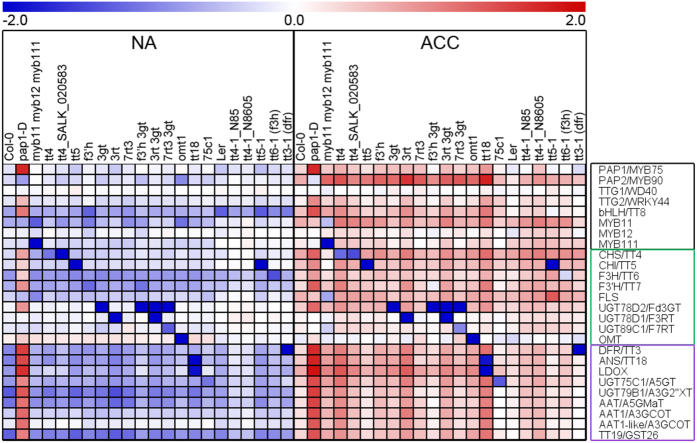
Relative transcript abundance (2^−ΔCt^) of flavonoid biosynthesis genes in mutant lines and the corresponding wild types under non-acclimated (NA) and cold acclimated (ACC) conditions. Mutant lines are sorted according to their positions in the flavonoid biosynthetic pathway. Data represent averages of three biological replicates per line and condition, log_10_ median transformed over all lines and both conditions for every gene with transcript abundance above median in red and below median in blue, as indicated by the scale bar. Genes encoding transcription factors are framed in black, flavonol biosynthesis genes in green and anthocyanin biosynthesis genes in purple (see also Suppl. Table 1).

**Figure 3:**
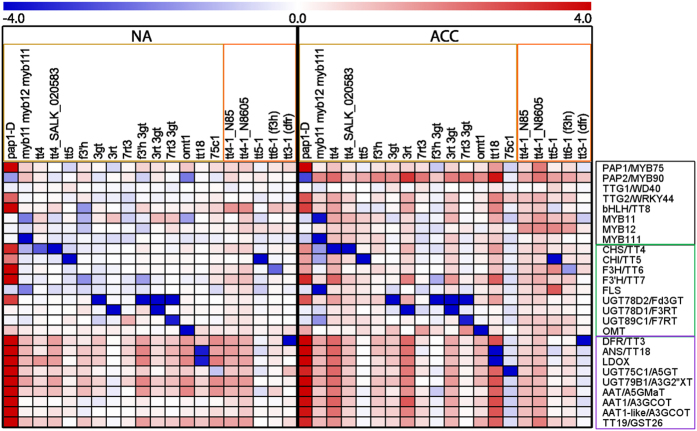
Log2 fold change of relative transcript abundance (2^−ΔCt^) of flavonoid biosynthesis genes in mutant lines relative to Col-0 or L*er* wild type under non-acclimated (NA) and cold acclimated (ACC) conditions. Mutant lines in Col-0 (yellow framed) and L*er* (orange framed) are sorted according to their positions in the flavonoid biosynthetic pathway. Data represent averages of three biological replicates per line and condition with higher relative expression in mutant lines compared to wild type in red and lower expression in blue, as indicated by the scale bar. Genes encoding TFs are framed in black, flavonol biosynthesis genes in green and anthocyanin biosynthesis genes in purple (see also Suppl. Table 1).

**Figure 4:**
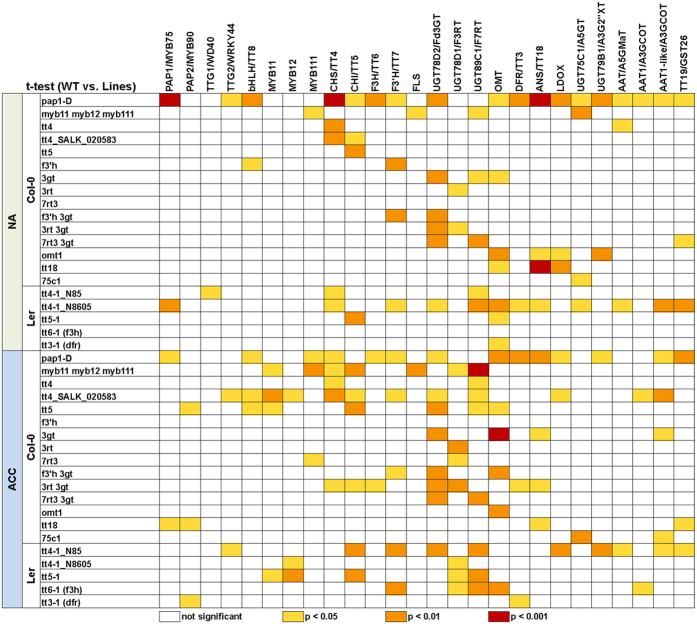
Statistical analysis of gene expression data (log_2_-fold change) shown inFig. 3. t-tests (two-sided with unequal variance) were used to determine the significance of expression changes between mutant lines and their wild types Col-0 or L*er* under non-acclimated (NA) and acclimated condition (ACC). p-values were corrected using the Benjamini-Hochberg procedure and color coded as indicated in the figure (see also Suppl. Table 4).

We used quantitative RT-PCR to characterize the effects of the mutations on transcript levels of 26 genes encoding transcription factors regulating flavonoid biosynthesis and enzymes in the flavonoid biosynthetic pathway (Fig. 2; Suppl. Table 1). As described in detail recently, this analysis revealed a strong cold-induction of the pathway at the transcript level, with the exception of *TTG1*/*WD40* and *OMT1*, which showed high constitutive expression^20^. The ko mutant lines generally showed a significant decrease in the abundance of the transcripts encoded by the affected genes compared to the wild type under both cold acclimated and non-acclimated conditions (Figs 3 and 4; Suppl. Table 4). Notable exceptions were the lines *f3*′*h, f3*′*h 3gt* and *myb11 myb12 myb111* in Col-0, and both *tt4-1* lines in L*er*. The T-DNA insertion in the *F3*′*H/TT7* gene is localized in the fourth exon immediately upstream of the 3′UTR region, rendering the design of qRT-PCR primers that bind beyond the T-DNA impossible. This led to the detection of partial transcripts with our primers binding in the second exon. The *MYB12* transcript in the triple mutant contains a single base pair deletion (*myb12-1f*) and is measurable by qRT-PCR, but results in premature termination of translation^32^. Similarly, the detected *CHS/TT4* trancripts in the *tt4-1* mutants in L*er* are nonfunctional due to point mutations^33^. It remains unclear why the expression of *UGT89C1/F7RT* was significantly reduced in the *7rt3 3gt* double mutant, but was almost unchanged in the *7rt3* single mutant line.

In general, mutations in the genes encoding enzymes had only minor effects on the abundance of all other investigated transcripts (Fig. 4; Suppl. Table 4). On the other hand, knock-out of the MYB11, MYB12 and MYB111 transcription factors resulted, as expected^14^, in strongly reduced expression of key flavonol biosynthesis genes such as *CHS*/*TT4, CHI*/*TT5* and *FLS*, especially after cold acclimation. The activation tagging line *pap1-D* showed high *PAP1/MYB75* transcript abundance under both non-acclimated and acclimated conditions and constitutive activation of down-stream genes encoding the transcription factors TTG2/WRKY44 and bHLH42/TT8, some flavonol and most anthocyanin biosynthetic enzymes. In addition, two of the four tt4 lines showed a significant upregulation of several of the genes encoding down-stream enzymes.

### Effects of mutations on flavonoid content

**Figure 5:**
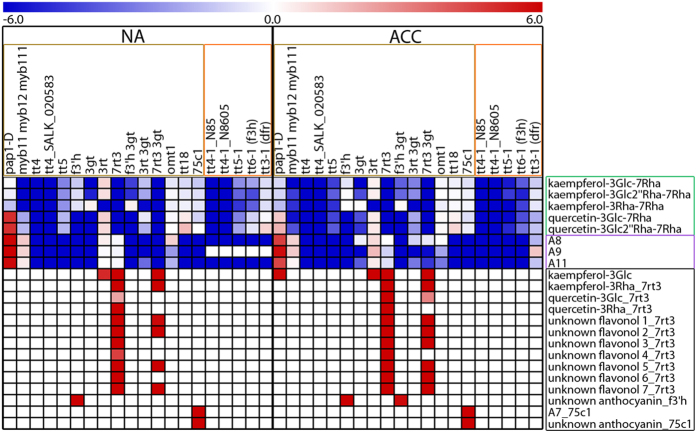
Flavonoid content (relative peak area) in mutant lines changed in flavonoid metabolism under non-acclimated (NA) and acclimated (ACC) conditions. Mutant lines in Col-0 (yellow framed) and L*er* (orange framed) are sorted according to their positions in the flavonoid biosynthetic pathway. Data represent averages of three or six (only Col-0 wild type and single mutants) biological replicates with higher flavonoid content in mutant lines in comparison to corresponding wild type in red and lower content in blue, as indicated by the scale bar. Central flavonols and anthocyanins are framed in green and purple, minor flavonoids and flavonols with unknown structure in black (see also Suppl. Table 2).

**Figure 6:**
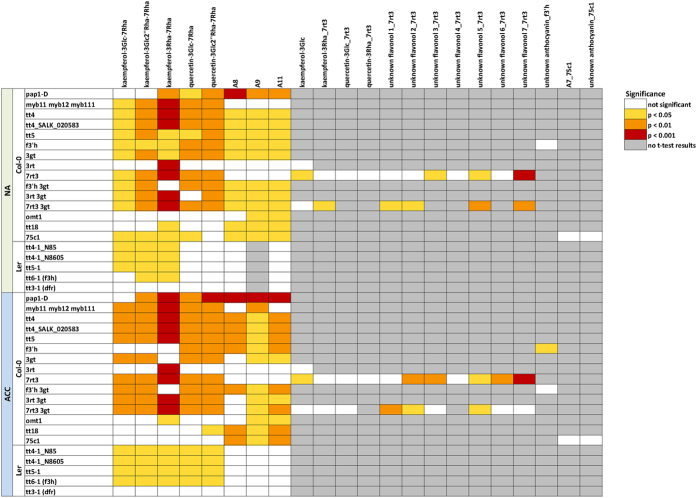
Statistical analysis of differences in flavonoid content between mutant lines and their wild types Col-0 or L*er* under non-acclimated (NA) and acclimated condition (ACC). t-tests (two-sided with unequal variance) were used to determine the significance of expression changes. p-values were corrected using the Benjamini-Hochberg procedure and color coded as indicated in the figure. Gray fields indicate that no t-test could be performed because the metabolite was undetectable in these samples (see also Suppl. Table 5).

We further evaluated by LC-MS analysis, whether the observed changes in gene expression in the mutant lines also resulted in corresponding changes in flavonoid content (Fig. 5; Suppl. Table 2). Three kaempferol and two quercetin derivatives, three major anthocyanins (A8, A9 and A11), four minor flavonols and one minor anthocyanin (A7) as well as seven unidentified flavonols and two unidentified anthocyanins could be quantified. A preliminary identification of these compounds based on MS/MS fragmentation analysis is provided in Suppl. Table 2. The significance of differences in flavonoid content between mutants and wild types (Fig. 6; Suppl. Table 5) was evaluated by t-tests corrected for multiple testing errors^34^. Changes in *Arabidopsis* flavonoid content during cold acclimation have been reported recently^20^ and will not be considered here.

Figure 5 shows that most mutations resulted in the absence or a strong reduction of major flavonoids, while only *pap1-D* showed a strong accumulation, particularly of anthocyanins, but also of the two major quercetin derivatives. Additionally, kaempferol-3Glc increased massively in the acclimated state in this mutant. On the other hand, *myb11 myb12 myb111* showed a strong reduction in the content of all flavonols, but not of anthocyanins. All four investigated *tt4* lines in both accessions completely lacked flavonoids, while the *tt5* and *tt6-1* lines, also affected in the initial steps of the pathway, still contained low, significantly reduced levels of flavonols and anthocyanins, particularly under cold acclimated conditions. Similarly, the lines *75c1* and *tt18* in Col-0 and *tt3-1* in L*er* were specifically reduced in anthocyanin content.

All other mutant lines showed more specific effects on the content of particular flavonoids. For example, the *f3*′*h* lines lacked all major quercetin and anthocyanin derivatives, whereas the *f3*′*h 3gt* line additionally showed reduced levels of glucosylated kaempferols that were also affected in line *3gt*, while *3rt* was specifically reduced in its kaempferol-3Rha-7Rha content. The line *7rt3* showed a strong reduction of all central 7-*O*-rhamnosylated flavonols. Noteworthy, *7rt3* and *f3*′*h* single and double mutant lines as well as *75c1* accumulated mutant-specific flavonol or anthocyanin derivatives (see Suppl. Table 2 for preliminary structure assignments) which might be evidence of compensatory effects. These findings are consistent with previous studies under normal (non-acclimated) growth conditions^4^,^7^,^9^,^31^,^35^,^36^,^37^,^38^.

### Effects of mutations on leaf freezing tolerance

**Figure 7:**
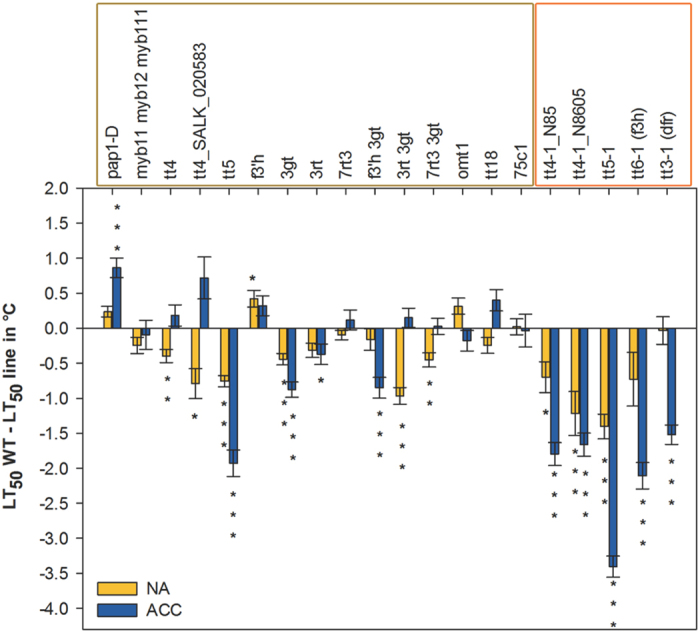
Freezing tolerance of all mutant lines (see Fig. 1) compared to the corresponding wild types, of non-acclimated and cold acclimated plants. Mutant lines in Col-0 (framed in yellow) and L*er* (framed in orange) are sorted according to their position in the flavonoid biosynthetic pathway. The bars indicate the differences in leaf freezing tolerance between wild type and mutant expressed as LT_50_ (temperature at which 50% electrolyte leakage occurred). Positive values indicate improved freezing tolerance, negative values impaired freezing tolerance compared to the respective wild type. Data are means from two to four independent plant cultures, each with four biological replicates per line and condition. Significant differences, evaluated by Welch’s unpaired t-test, are marked by asterisks: *p < 0.05, **p < 0.01, ***p < 0.001.

To assess whether flavonoids play a functional role in leaf freezing tolerance, we compared wild type and mutant lines both before and after cold acclimation (Fig. 7), using an electrolyte leakage assay^39^. Only the *pap1-D* line showed significantly improved freezing tolerance under acclimated conditions, which can be attributed to the accumulation of quercetin and anthocyanin derivatives, while all ko lines showed either reduced freezing tolerance or were not significantly affected.

Mutants in the three earliest steps of the pathway (*tt4, tt5, tt6*) which showed a massive reduction in both flavonols and anthocyanins, exhibited a strong reduction in freezing tolerance in the L*er* background, in particular after cold acclimation. Interestingly, while the *tt5* mutant behaved similarly in Col-0, this was not found for the *tt4* mutants in this accession, which only showed reduced freezing tolerance in the non-acclimated state. On the other hand, the *tt3-1* mutant in L*er* showed a significant reduction in cold acclimated freezing tolerance, although only small, non-significant differences in flavonoid content could be detected compared to the wild type (Figs 5 and 6).

No changes in freezing tolerance were observed in mutants where only anthocyanin (*tt18, 75c1*) or only flavonol contents (*myb111 myb12 myb111*) were reduced. However, when all flavonoids were strongly reduced (*tt5, tt6-1* and *3rt 3gt*) freezing tolerance was impaired at least under one condition. The reduction of all 3-*O*-glucosylated flavonoids in 3*gt* reduced freezing tolerance under both conditions, while a complete lack of only kaempferol-3Rha-7Rha in *3rt* resulted in slightly impaired freezing tolerance only under non-acclimated conditions. The absence of all major flavonols in *7rt3* accompanied by the occurrence of flavonols with unknown structure at unchanged anthocyanin levels had no impact on freezing tolerance. The additional reduction of anthocyanins in the *7rt3 3gt* double mutant led to impaired non-acclimated freezing tolerance. In addition, a complete lack of quercetin and anthocyanin derivatives with unchanged kaempferol levels in *f3*′*h* had no significant effect on freezing tolerance. The *f3*′*h 3gt* double mutant line was additionally reduced in kaempferol-3Glc-7Rha and kaempferol-3Glc2″7Rha-7Rha and therefore only unaffected in the content of kaempferol-3Rha-7Rha resulting in impaired freezing tolerance after cold acclimation.

### Effects of mutations on primary metabolism

Cold acclimation is accompanied by a massive accumulation of many primary metabolites such as sugars and amino acids that may act as compatible solutes to improve cellular freezing tolerance^27^,^28^. We therefore checked whether the mutations in secondary metabolism had additional effects on primary metabolism. We compared the relative pool sizes of 114 primary metabolites (34 organic, amino and hydroxyl acids, four N-compounds, six P-compounds, 15 sugars and 55 unknown putative primary metabolites) measured by GC-MS (Suppl. Table 6) between wild type and mutant plants under both non-acclimated and acclimated conditions. This analysis showed only very little effect of mutations in the flavonoid biosynthetic pathway on primary metabolism under either condition, with only eight significantly different pool sizes among 114 metabolites in 40 comparisons (Suppl. Table 6) in agreement with an earlier study that was conducted only under non-acclimating conditions^4^.

## Discussion

Comprehensive characterization of the flavonoid mutants with respect to their freezing tolerance, gene expression and metabolite profiles indicates a role of flavonoids in freezing tolerance and cold acclimation. Previous work has implicated flavonoids in the defence against ROS (e.g. ref. 15), although the importance of flavonoids for ROS scavenging *in vivo* has been questioned^40^. This ROS-scavenging activity has been invoked mainly for plant chilling tolerance, i.e. tolerance against low temperatures above 0 °C, while here we investigated the tolerance of plants against freezing, i.e. the stresses associated with apoplastic ice formation at sub-zero temperatures. A clear physiological role for flavonoids, on the other hand, has been established for their UV-B screening function^18^,^30^,^41^. A function of flavonoids in plant freezing tolerance has been hypothesized in *Arabidopsis* due to the correlation of both metabolite and transcript abundances with freezing tolerance^19^,^20^,^23^, but a clear causal relationship had not been established previously.

**Figure 8:**
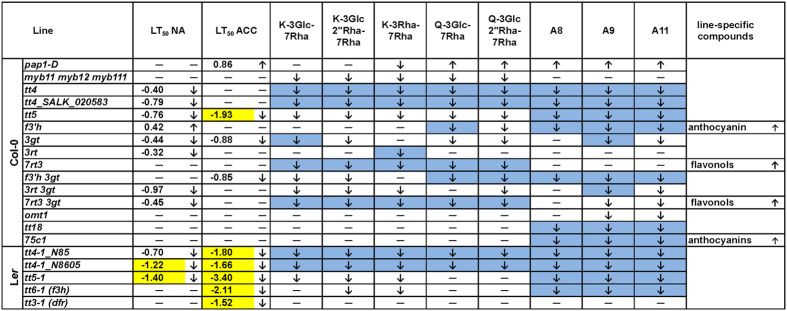
Overview of the effects of all investigated mutations on non-acclimated (LT_50_ NA) and acclimated freezing tolerance (LT_50_ ACC) and on flavonoid content. Significantly changed LT_50_ values are shown (LT_50_ wild type – LT_50_ mutant) and decrease (↓), increase (↑), and no changes (—) are indicated. Changes in LT_50_ of more than 1 °C are highlighted in yellow. Significantly decreased (↓) or increased (↑) contents of kaempferols (K), quercetins (Q), anthocyanins (A) and mutant-specific flavonoids are indicated for both NA and ACC conditions combined. Complete absence of a compound is indicated in blue.

In contrast to the overexpression line *pap1-D* with improved freezing tolerance, most of the ko mutant lines showed reduced freezing tolerance. In general, the complete loss or strong reduction of flavonoid compounds resulted in impairment of freezing tolerance or in the accumulation of other known or unknown flavonoids with potential compensatory effects, while the accumulation of predominantly quercetin and anthocyanin compounds in the *pap1-D* mutant enhanced freezing tolerance (see Fig. 8 for a summary of these results). This is in good agreement with our previous finding that the content of all three quercetin derivatives and of the anthocyanins A5 and A9 were correlated with acclimated freezing tolerance in a collection of Arabidopsis accessions^20^. Interestingly, the reduction of either flavonols or anthocyanins did not result in a decline in freezing tolerance, indicating that the two groups of compounds may have at least partially redundant functions. As the presence of kaempferols, flavonols in general or anthocyanin derivatives preserved freezing tolerance, the combined contribution of a set of different compounds is more likely than the specific action of a particular molecule.

Differences in flavonoid composition between Col-0 and L*er* have been reported previously for both plants exposed to non-acclimating and cold acclimating conditions^20^,^42^. The effects of mutations in the flavonoid biosynthesis pathway on freezing tolerance were generally more pronounced in the L*er* than in the Col-0 background, suggesting that the contribution of flavonoids to freezing tolerance may be genotype-dependent. This is supported by the fact that *tt3-1* in L*er*, with only a moderate reduction in anthocyanin levels, was affected in its acclimated freezing tolerance, while *tt18* and *75c1* in Col-0, with much stronger reductions in anthocyanin contents, were not affected. In addition, the L*er tt5-1* and both *tt4-1* lines were more strongly affected in their freezing tolerance than the corresponding lines in Col-0. This accession effect is particularly striking for the *tt4* mutants, which showed a significant reduction in anthocyanin content after cold acclimation in Col-0, but not in L*er*, while freezing tolerance was only reduced in L*er* (compare Fig. 8). The physiological basis of this difference between the accessions is currently unclear but it strongly underscores the fact that plant freezing tolerance is a quantitative trait that is based on the concerted action of many proteins and metabolites. This makes it difficult to estimate the contribution of single components to the total phenotype. For example, while raffinose content correlates strongly with acclimated freezing tolerance in *Arabidopsis* accessions, a ko mutant in the raffinose synthase gene that is completely devoid of raffinose shows no measurable impairment in freezing tolerance^43^.

Due to their antioxidant activity, a possible protective mechanism of flavonoids during freezing may be the scavenging of ROS^44^. In our experiments, however, this is rather unlikely, as freezing of leaves was performed in the dark to exclude ROS formation in the chloroplast. In addition, the cold acclimation treatment was performed at a significantly lower light intensity than growth under control conditions, also reducing ROS accumulation^29^. Flavonoids could also modulate stress response pathways through phytohormones as they negatively regulate auxin transport^45^ and jasmonate levels^46^. However, we find such a pleiotropic effect unlikely, as plant growth, development and primary metabolism were unaffected in all mutants used in this study.

The direct protection of membranes and/or proteins from freezing damage are other possible mechanisms through which flavonoids could increase leaf freezing tolerance, in particular since flavonoids have been localized in the vacuole, nucleus and chloroplast, which may all be protected by flavonoids during freezing^47^. Membranes have long been recognized as major sites of freezing injury^48^ and soluble cold-induced proteins that specifically stabilize membranes during freezing in *Arabidopsis* have been characterized^49^,^50^. A similar function of flavonoids seems possible as they can partition into both model and plant membranes *in vitro* and influence their stability^51^. In addition, enzymes can be inactivated when *Arabidopsis* leaves are frozen to temperatures that also induce electrolyte leakage and the inactivation of photosynthesis^50^. Flavonoids are able to stabilize proteins *in vitro* by preventing their aggregation^52^,^53^,^54^. Clearly, further research is needed to understand the functional mechanisms that link flavonoid content to plant freezing tolerance.

## Methods

### Plant material

The mutant lines used in this study included one activation tagging line and 13 T-DNA insertion lines of which nine were single, three double and one triple gene knock-outs in Columbia-0 (Col-0). Five mutants in Landsberg *erecta* (L*er*) were generated by ethylmethansulfonate (EMS), fast neutron or x-ray mutagenesis and one line in Col-0 by carbon ion induction. In addition we used one activation tagging line. Details to all lines are given in Fig. 1. Plants were grown in a greenhouse at 16 h day length with light supplementation to reach at least 200 μE m^−2^ s^−1^ and a temperature of 20 °C during the day, 18 °C during the night until 42 days after sowing (non-acclimated plants). For cold acclimation, plants were transferred to a 4 °C growth cabinet at 16 h day length with 90 μE m^−2^ s^−1^ for an additional 14 days^23^,^55^.

### Determination of leaf freezing tolerance

Freezing damage was determined as LT_50_ (temperature of 50% electrolyte leakage) from electrolyte leakage measurements after freezing of detached leaves to temperatures ranging from −1 to −21 °C at 4 °C h^−1^ as described in detail in previous publications^39^,^56^. Per line and condition four temperature curves were analyzed in each experiment, using leaves from three to six individual plants each. Due to the number of mutant lines, several experiments had to be performed, which each contained samples from the respective wild type. Data were collected for every mutant line from two to four independent sowings.

### qRT-PCR analysis of gene expression

Quantitative RT-PCR was performed according to ref. 20, where also the sequences of all primers can be found. Ct values for the genes of interest were normalized by subtracting the mean Ct of four reference genes and averages of three biological replicates were determined (Suppl. Table 1).

### LC-MS analysis of flavonoids

Secondary metabolite profiling by LC-MS was carried out as described recently^20^,^57^. Peak identification and annotation was performed with Xcalibur (Thermo Finnigan) based on previously published reference data^4^,^58^,^59^. Relative peak areas representing mass spectral ion currents were normalized to fresh weight and peak area of the internal standard. The data represent the averages of three or six (Col-0 wild type and single mutants) biological replicates (Suppl. Table 2). The relative peak area obtained for kaempferol-3Glc-7Rha also represents a minor contribution from quercetin-3Rha-7Rha, as these compounds were not completely separated.

### Primary metabolite profiling by GC-MS

Polar metabolites were extracted from100 mg frozen plant material and analyzed by gas chromatography/electron impact ionization-time of flight mass spectrometry (GC/EI-TOF-MS) as described in detail recently^60^. For every metabolite, relative mean intensities were calculated for each genotype as the average of five biological replicates (Suppl. Table 3).

## Additional Information

**How to cite this article**: Schulz, E. *et al*. Flavonoids are determinants of freezing tolerance and cold acclimation in *Arabidopsis thaliana. Sci. Rep.* **6**, 34027; doi: 10.1038/srep34027 (2016).

## Supplementary Material

Supplementary Table 1

Supplementary Table 2

Supplementary Table 3

Supplementary Table 4

Supplementary Table 5

Supplementary Table 6
